# Future Risk of Bovine Tuberculosis (*Mycobacterium bovis*) Breakdown in Cattle Herds 2013–2018: A Dominance Analysis Approach

**DOI:** 10.3390/microorganisms9051004

**Published:** 2021-05-06

**Authors:** Andrew W. Byrne, Damien Barrett, Philip Breslin, Jamie M. Madden, James O’Keeffe, Eoin Ryan

**Affiliations:** 1 One-Health Scientific Support Unit, National Disease Control Centre (NDCC), Department of Agriculture, Food and the Marine, Agriculture House, D02 WK12 Dublin 2, Ireland; damien.barrett@agriculture.gov.ie; 2 Ruminant Animal Health Division, Department of Agriculture, Food and the Marine, Backweston Co., W23 X3PH Kildare, Ireland; philip.breslin@agriculture.gov.ie (P.B.); james.okeeffe@agriculture.gov.ie (J.O.); 3 Centre for Veterinary Epidemiology and Risk Analysis (CVERA), School of Veterinary Medicine, University College Dublin, Belfield, D04 V1W8 Dublin 4, Ireland; jamie.madden@ucd.ie

**Keywords:** TB, one health epidemiology, cattle, Ireland

## Abstract

Bovine tuberculosis (bTB) remains a significant endemic pathogen of cattle herds, despite multi-decadal control programmes being in place in several countries. Understanding the risks of future bTB breakdown (BD) and the associated characteristics of herds and index breakdowns could help inform risk categorisation. Such risk categories could then contribute to tailored management and policies. Here, we estimated the future risk of herd BD for the cohort of herds that were derestricted during 2013 in Ireland using multivariable logit regression models, with a dominance analysis approach. One third of herds that were derestricted in 2013 experienced a breakdown during the follow-up five year period (1469/4459; 33%). BD length was a significant predictor of future risk, primarily driven by long BDs > 230 days relative to short BDs < 130 days (OR 95%CI: 1.157–1.851), as was having had a previous BD (OR 95%CI: 1.012–1.366). Herd-size was the dominant predictor of future risk (accounted for 46% of predicted variance), suggesting significant increase in risk of future breakdown with increasing (log) herd-size (OR 95%CI: 1.378–1.609). There was significant spatial variation in future risk across counties, and it was the second most dominant predictor of future risk (25% of predicted variance). The size of index breakdowns was not a strong predictor of future risk over a 5-year period. These findings can inform a risk-based policy development.

## 1. Introduction

The *Mycobacterium tuberculosis* complex (MTBC) includes *Mycobacterium bovis*, the causative agent of bovine tuberculosis (bTB). This disease has a significant impact on livestock, on a global scale, with veterinary health, public health and financial impacts [1,2,3]. Bovine TB is a significant One Health challenge throughout much of the world, and, recognizing this, a multi-institute joint initiative to reduce zoonotic bTB infections globally has been launched [4,5]. The pathogen has remained stubbornly persistent despite many decades of control programmes in several developed countries [1,3,6,7]. Developing an evidential base for policy development and appropriate interventions is central to advancing effective programmes in countries that are actively trying to reduce, and ultimately eradicate, bovine tuberculosis in both their domestic stock and wildlife/environment [7,8,9,10].

One such country is Ireland, where bTB remains a priority endemic disease, affecting cattle herds [7,9,11]. A national bovine eradication scheme was established in 1954 [12]. All herds in Ireland are subject to an annual test using the SICTT (Single Intradermal Comparative Tuberculin Test), with follow-up risk-based testing where appropriate. Test-positive animals (known as ‘reactors’) are slaughtered, and trade/animal movement restrictions are imposed until the herd regains its officially TB-free status through passing two herd tests 60 days apart. Routine surveillance for suspected post-mortem TB lesions is carried out on all cattle, and where such cases are confirmed, the herd of origin is restricted, and follow-up testing conducted. Once bTB is identified within a herd, risk-based area-focused control measures may be also put in place, including a programmes of contiguous herd testing. Despite these control programmes being in place, bTB has persisted [7]. There are several reasons why this may have been the case (see Allen et al. [7] for discussion), but key issues include the presence of a wildlife reservoir of infection (in Ireland, the main reservoir species is the European badger, *Meles meles*), the performance of available diagnostic tests, and the difficulty in clearing infection from within herds once introduced. These challenges are not unique to Ireland, and such limitations to progress have been identified in several other countries including the UK [8], Spain [6], France [13], and New Zealand [10], for example.

Recrudescence of infection is a known feature of bTB epidemiology in the UK and Ireland [14,15,16,17,18,19], and elsewhere [20,21,22,23]. Herds that experience a breakdown (that is, when one or more animals present with evidence of bTB via antemortem tests or postmortem surveillance) tend to be at a greater risk of future breakdown. This recurrence of breakdown risk has partly been attributed to the ineffectual clearance of all infected hosts during a breakdown, sometimes called a ‘hidden burden’ of infection [24]. Truly infected animals may be missed during routine testing, as the standard interpretation of the SICTT exhibits moderate sensitivity, estimated to range from 50–70% (but very high specificity; >99%; [25,26,27]). Repeated testing of individuals, as used prior to the release of herds from restriction, could improve diagnosis at herd-level, but may miss early-stage or late-stage infection in cattle. Ancillary gamma interferon testing is being used to identify additional infected animals [28].

Gaining an understanding of the risk of future breakdown and ranking of risk factors are vitally important for the efficient use of resources attributed to control. Despite previous research on recrudescence in Ireland and elsewhere, there were several reasons we undertook the current study:

1. The Irish TB programme is evidence based (see More [9]), it relies on continual engagement with research endeavours [29], and it requires policies to be based on the most up-to-date data available.
2. The epidemiologic situation has changed in recent years, with the study period representing an historic low incidence in TB in Ireland (2013–2018; [9]).
3. There was a significant change in the structure of Irish farming post lifting of milk quotas in 2015, with significant shift towards dairy farming, associated with increasing milk production and increasing herd-size. For example, from 2008 to 2013, an average dairy herd from a representative sample increased the herd-size by 12%; higher performing (top quartile) herds increased the herd size by 37% [30]. It was unknown whether such change might impact risk factor analyses for recurrent breakdowns.
4. Importantly, we apply for the first time a technique (dominance analysis) to rank factors in terms of the importance to affecting future risk. Dominance analysis seeks to explore the variance explained by regression models (variance decomposition; see Grömping [31] for a discussion) and ranks predictors in terms of their importance to the global model [32]. The application of dominance analysis allowed us to partition the variation explained by our model to inform our understanding of which factors may be the most useful to target for policies aimed at reducing future recrudescence.

Therefore, our primary objective was to examine factors that affect future risk of bTB breakdown, and our secondary objective was to rank these factors in terms of their importance. The results will inform the continuing development of TB eradication policy, providing an evidence-based underpinning for enhancing risk-based controls.

## 2. Materials and Methods

A cohort, herd-level quantification of future risk was undertaken using a “breakdown level” dataset (see [33] for more). This breakdown dataset was constructed such that each line of the data was a discrete breakdown initiated by the disclosure of standard reactors at test or by post-mortem disclosure of infected animals (i.e., TB lesions disclosed during routine slaughter; J. Madden, pers. com.). Note, we included “unconfirmed” breakdowns, that is irrespective of whether reactors were confirmed via pathology (TB-like lesions being present) and/or laboratory confirmation. Breakdowns ended when herds were derestricted (free to trade) following bTB programme rule sets (e.g., two clear herd tests, 60 days apart) and in compliance with EU bTB regulations. The primary aim of this study was to fit a model to estimate the future risk of bTB breakdowns for all herds within Ireland that were derestricted (i.e., had a breakdown end), within 2013.

The outcome variable was a binary categorisation, where ‘1′ indicated a 2013 derestricted herd that experienced a breakdown during the follow-up period; a ‘0′ indicated a 2013 derestricted herd that did not experience a herd breakdown period during the follow-up period.

The follow-up (risk) period was from derestriction to 31 December 2018. As derestriction date was variable, the follow-up period for each herd was limited to 5 years post derestriction date (i.e., <1826 days post-derestriction).

The factors modelled were chosen as they have been previously identified as key modulators of bTB herd-level risk [8,34] and were considered important from a policy perspective. Independent variables included:

- Breakdown size (number of reactors) during the index test case;
- Post-mortem confirmation (visible lesion in reactors at slaughter);
- Breakdown length (days);
- Herd history (breakdown during the previous 5 years);
- Herd-size at derestriction;
- Herd-type (beef (rearing/finishing), dairy, suckler (non-dairy breeding herd), and other), as designated on the Animal Health Computer System (AHCS; DAFM);
- County.

### 2.1. Model Building and Assessment

The outcome was a binary outcome indicating whether a derestricted herd had one or more breakdowns in the follow-up period of 5 years. This dichotomous response variable was modelled using a logit distribution. Initially, a series of univariable logistic regression models were run to assess the relationship between the outcome and each predictor, respectively (to estimate unconditional associations).

All independent variables were associated with this outcome within a multivariable linear model (logistic regression). No model selection procedures were employed, as there were *a priori* good reasons to assume that all variables offered to the model were associated with the outcome (i.e., both the biological understanding of bTB risk in Ireland and the findings from previous research). Furthermore, we wanted to estimate the possible effect size of particular variables in terms of future risk, irrespective of whether they were ‘significantly’ associated, due to their utility in characterising BDs for policy development purposes. Finally, as there was the potential for correlations within the predictor set, we felt that modelling the outcome in a saturated model was a sensible choice [35].

The model fit was assessed using the Hosmer-Lemeshow test. The discriminatory ability of the model was assessed using the Area Under the ROC curve (AUC), with a conventional interpretation for a predictive model that was poor if AUC < 0.6; fair if AUC: 0.6–0.7; good if AUC 0.7–0.8; and excellent if AUC > 0.8. The model’s predictive performance (i.e., sensitivity, specificity) was assessed at different cut-points: 0.40, 0.50, and 0.60. We also estimated the cut-point if specificity of the model was fixed to 80%, via iterative recalculation.

All predictors were fitted as fixed effects, except for ‘county’ (i.e., a spatial fixed effect), which was included or omitted in competing models.

### 2.2. Dominance Analysis

A dominance analysis was undertaken with the final fully saturated (fixed effect) model [36]. The original dominance models focused on Gaussian linear regression models [32]; however, developments have allowed for extensions to include the application of these approaches to categorical data [37], including logit models [36] and multivariable modelling space [38]. The analysis is an ensemble approach, fitting multiple models based on the composition of the predictors (right hand side of the regression model), keeping and dropping each combination of the factors in and from a collection of models (i.e., all combinations fitting). Dominance analysis then explores the predictors in a linear model with three parameters: general dominance, conditional dominance, and complete dominance. Throughout, the comparison of models is based on a metric of model fit, and a generalised least squares model R^2^ is used, with logistic regression McFadden R^2^ as a suggested alternative [36].

Complete is the strongest evidence of dominance and indicates where a predictor is dominant over another predictor in all possible combinations of independent predictors tested (all subsets; [38]), i.e., if independent variable *X* has a larger incremental contribution to model fit than independent variable *Y* across all possible comparisons, independent variable *X* “completely dominates” independent variable *Y* [39]. Conditional dominance is measured as the mean greater incremental improvement to model fit across a subset of models of size *k*, for which the predictor variable is included. If the average contribution for predictor *X* for this subset of models is greater than predictor *Y*, then *X* is considered conditionally dominant relative to *Y* [38]. Finally, the weakest evidence of dominance is the general dominance, which is the overall mean of all conditional values, which, together, add to the fit statistic. Azen and Budescu [38] describe it as “the general measure [which] represents the average difference in fit between all subset models (of equal size) that include *X* and those that do not include it”. This value can then be standardised to allow interpretation of the partial contribution of the predicted variance. Murray and Conner [40] have recommended the Dominance Analysis approach as the best for ranking variables, based on a simulation comparison of six competing methods to quantify variable importance.

For each predictor in the full model, the general, conditional, and complete dominance based on McFadden R^2^, across all possible combinations of the ensemble of 127 regression models, were undertaken (n predictors = 7). The general dominance values were standardised relative to 100%, allowing for the interpretation for the maximal variation explained by each predictor in the model [39]. Note, we did not fit the relative weights (epsilon) heuristic model, which, despite the advantage of its computational speed, is considered to give biased results.

All analyses were undertaken in Stata/MP 15.1.

## 3. Results

There were 4530 derestrictions (i.e., they experienced a bTB breakdown with an end date during 2013) during the year 2013, from 4,459 herds. A total of 71 herds had two derestrictions during 2013, in which case the last derestriction within 2013 was used in this analysis (i.e., one observation per herd). Approximately one third of derestrictions in 2013 experienced a breakdown during the follow-up period (32.9% 1469/4459). A summary of the herds and their characteristics is presented within Table S1. Overall, the cohort composition had more herds categorised as sucklers (45.0%; *n* = 2007), compared with dairy, beef, or other (Table S1). County Cork was associated with most herds within the dataset (*n* = 596; 13.4% of all herds), followed by Co. Clare (*n* = 297; 6.7%) and Co. Galway (*n* = 288; 6.5%). It should be noted that only 28 herds (0.6%) were associated with Co. Dublin, and therefore should be treated with caution. The average herd-size was 101.04 (SD: 102.88) animals but was affected by a small number of very large herds (>1000), resulting in a median herd-size of 70 animals. There was a significant relationship between herd-type and herd-size (negative binomial regression herd-size outcome: *P* < 0.001), with the mean herd-size count being significantly larger in dairy herds relative to all other herd-types. For example, the mean herd-size for dairy herds was 175.82 (SD: 112.76) relative to 71.20 (SD: 83.26) for beef herds (Incidence Rate Ratio (IRR): 2.469; 95%CI: 2.297–2.654). Therefore, we expected some confounding when herd-type and herd-size were entered into the same model.

### 3.1. Univariable Associations

Univariate model results are presented in Table S2. These unconditional models suggested significant increasing risk of a future breakdown within 5 years with increasing reactors, BD length, BD history, and herd-size. Dairy herds had a significantly greater risk of future BD relative to beef (finishers), other, or suckler herd-types.

### 3.2. Multivariable Model Results

Two models were fitted to the data, an aspatial model (county omitted; Table S3) and a full, ‘saturated’, model including county as a fixed effect (fe). As the parameter estimates across models were broadly similar, we will here concentrate on the full saturated fe model.

The final model was composed of 4459 observations (Table 1). Saturated, multivariable logit regression models revealed that, with this cohort and follow-up period, there was no significant increase in risk with the detection of a lesion at slaughter (OR: 1.008; 95%CI: 0.844–1.202) or with the increasing number of reactors disclosed (categorical variable; χ^2^(DF: 5) = 6.75; Prob > χ^2^ = 0.240). Herds breaking down with zero reactors appeared to have greater future risk, relative to single reactor breakdowns (OR: 1.224; 95%CI: 1.018–1.471).

Duration was a significant predictor, primarily driven by long BDs > 230 days relative to short BDs < 130 days (OR: 1.489; 95%CI: 1.187–1.869). Having a previous BD (of any size) was positively associated with future risk (OR: 1.200; 95%CI: 1.030–1.399). There was significant increase in risk of future breakdown with increasing (log) herd-size (OR: 1.487; 95%CI: 1.376–1.607; Figure S2). Herd-type significantly explained the variation in outcome, primarily attributable to reduced risk for suckler and other herds, relative to beef; controlling for herd-size (confounder), there was no significant variation between dairy and beef (beef vs. dairy future risk: OR: 0.874; 95%CI: 0.707–1.081). There was significant spatial variation in future risk across counties (*p* < 0.001; Table 1).

This full model had a fair discriminatory ability, with an AUC 0.67 (95%CI 0.65–0.68; see Figure S1 for ROC curve). At a cut-point of 0.4, SE was 44.3%, while SP was 77.7%; at a cut-point of 0.5, SE was 17.2%, while SP was 93.3%; at a probability cut-off of 0.60, the SE of the model was only 4.1%, while SP was high, at 98.9%. Fixing SP to 80.0% yielded a probability cut-off of 0.411 from model predictions, giving the sensitivity of the model of 41.0%; 67.2% of the herds were correctly classified with the model at this cut-off (Table S4).

### 3.3. Dominance Modelling

The general dominance statistic values ranked (log) herd-size as the most important variable contributing to the model (Table 2). The standardised dominance value suggested that herd-size alone contributed to 46.28% of the predicted variance in the model. This was followed by county (24.82%) and herd-type (12.65%). The conditional dominance results suggested that herd-size was conditionally dominant over each other independent variable, respectively, across all subset models (see Supplementary Material 1 for detail). The complete dominance designation suggested that herd-size was completely dominant over all other predictors; county was completely dominant over all predictors with the exception of herd-size. Herd-type was completely dominant over lesion presence, reactor numbers, and previous history; it was non-dominant relative to BD length (duration), and it was dominated by herd-size and county. The highest ranked BD characteristic was BD (duration), which only contributed to 7% of the fit statistic, and only completely dominated lesion presence and previous history.

## 4. Discussion

The present study updates previous work and further explores bovine tuberculosis future risk in Ireland (e.g., [14,15]). Our work has found that future risk is associated with characteristics of the index breakdown, though these features appear to explain limited variation in future risk based on dominance analysis. While population attributable fraction (PAF) has been used in the past to assess the proportion of cases in a population attributable to bTB risk factors [41], the current study for the first time applies a robust variable importance algorithm [40] to rank bTB predictors.

Herds with index breakdowns > 1 standard reactor appear to have greater future risk over a 5-year timeline than single reactor breakdowns, though this effect was non-significant in our multivariable models (significant in the univariable model). Previous work found that breakdown size in the index case could be a predictor of future bTB risk in Ireland [42,43] and elsewhere (e.g., [18,20,44]); our non-significant result could relate to an improved follow-up on larger breakdowns, or it could be related to methodological differences between the studies (case/cohort definitions; 3- vs. 5-year follow-up; survival vs. logistic model). In the present study, ‘zero’ reactor breakdowns appeared to exhibit greater future risk than single reactor index breakdowns; such ‘zero’ reactor breakdowns are caused by the disclosure of lesions found in a routine slaughter, but with no follow-up reactors found at the index test [33]. The presence of a lesion detected amongst SICTT reactors at slaughter, however, appeared to have little association with future risk. Having a breakdown within the previous five years of the index breakdown is associated with increased risk in the present study, mirroring the findings in Spain, where residual infection from previous outbreaks was the most frequent assigned cause of bTB breakdowns [21]. Breakdown length was the ‘best’ index breakdown predictor of future risk, as assessed using a dominance analysis, with long breakdowns (>230 days) having significantly greater risk of future breakdown relative to short breakdowns (<130 days). In Northern Ireland, Doyle et al. [19] found that total time restricted in the previous five years was a significant factor in recurrence risk.

By far, the most important variable associated with future risk in our model was herd-size (Figure S2). The standardised dominance statistic was 46%, with county being the second most important variable at 25%. Herd-size is consistently found to be a significant predictor of bTB risk across multiple studies in Ireland and elsewhere [7,8,34,45] and is therefore not surprising to emerge as the most important factor here also for future risk. However, we do not know what aspects of large herds may be driving this phenomenon—for example, large herd often have larger geographic footprint, exposing them to more neighbours [34], they may have more intensive farming approaches that may impact the immunocompetency of the cattle, or they may differ in trading practices (or may be at-risk due to trade network position; [46]). Unravelling the relationship between the metrics of farming intensity, farm structure and configuration (surface area, number of land parcels), herd size, and TB risk may be a fruitful avenue for future research [47,48,49].

While examining the transmission of infection between hosts, spatial correlation is an important consideration that is often ignored in bTB regression models. To account for this, we have included herds’ county to control for broad scale spatial variations. County was a significant contributor to future risk, though this trend masks considerably within county variation across herds (Figure S3). Although useful, we acknowledge the limitations of using this approach; primarily that Irish counties represent a large spatial region, themselves [16] containing different spatial distributions of disease risk. However, the focus of this work is on a dominance analysis and not on spatial regression models which require the inclusion of fully specified spatial random-effects, and which are being examined in detail by our co-authors in another ongoing bTB project (Madden et al. pers. comm.). Geographic variation in risk may be attributable to variation in exposure to wildlife (e.g., [50]), it may be related to the disease/risk management of herds across regions [16], or it may be related to other environmental factors [51].

Herd-type was significant in our final model. In Spain, Alvarez et al. [52] found that dairy herds were associated with increased within-herd transmission, and the time to recovery after an outbreak significantly reduced relative to other herd types. Other research has found that dairy herds have increased risk of breakdown recurrence [18,20], but this finding is not consistent across studies [19,42]. In the present study, there was little difference in the final multivariable model between the major farming types, dairy versus beef finishing herds. Suckler and ‘other’ herd types were at significantly reduced future risk of breakdown relative to beef finishing or dairy herds. Suckler herds were, on average, smaller herds, which may have contributed to their reduced future bTB risk relative to other herd types. They also have a different age-profile to dairy and beef finishing herds—calves move on to beef finishing herds or slaughter—and it is known that increasing age can be an animal-level risk factor for bTB risk [53]. Relative to suckler herds, dairy herds have an older animal profile and larger average herd sizes. Immune suppression via physiological stress in dairy animals may also be a factor impacting bTB risk [54]. Beef finishing herds include designated controlled beef finishing units (CBFU), colloquially known as “feedlots”, which have been found to be of significant increased risk of prolonged breakdowns [49]. Such herds can maintain the inward movement of animals, even during active breakdowns, as animals go straight to slaughter after short periods of fattening and are therefore deemed to be low risk for further transmission. In Northern Ireland, strain diversity of *M. bovis* across bTB breakdowns in beef finishing units was linked to inward animal movements from a wide geographical area [55].

A limitation of the present study was the omission of animal level data, which may have given an insight into how the composition of herds affect future risk. Tratalos et al. [56] have recently characterised the spatial and network components of animal-level Irish cattle movements, highlighting the scale and scope of such moves. However, this was deemed outside of the scope of the present study, as the purpose was to investigate and rank selected herd-level risk factors, and their performance for modelling herd risk.

Our findings provide an evidence base to underpin policy development on bovine TB eradication, enabling the recent data on risk factors to inform risk-based control measures. This work is of wider relevance to other countries with similar bovine TB challenges, as it describes a methodological approach that can help inform prioritisation efforts on tackling risk factors and the identification of the hierarchy of risk factors, which can inform *a priori* assumptions about epidemiological risks in country-specific analyses.

## 5. Conclusions

Our work highlights two points we believe are important to bTB research: 1. Previous findings are not immutable and therefore there is a value in re-examining relationships for evidence-based policy formation, especially during times of change to farming within states; and 2. risk factor ranking is a useful approach to help identify what policies might be most impactful in terms of disease control. In conclusion, in contrast to the previous research carried out in Ireland, the size of the index breakdowns was not a very strong predictor of a future five-year risk during this study period. Breakdown length and prior history were associated with future risk, but they explained a far less variation than herd-size, county, and herd-type, respectively. This study introduced a novel predictor weighting approach to bTB modelling in Ireland, which revealed how herd size is an extremely important characteristic of breakdown recurrence risk. This is important from a policy perspective, as farm structure is changing in Ireland in response to the changing of market and regulatory environments (e.g., dairy expansion in Ireland). Trading off herd size increase against disease risk will be an important policy challenge for bTB control in Ireland going forward.

## Figures and Tables

**Table 1:** Full multivariable model with spatial fixed effect (county) of the 5-year future risk of bTB breakdown for the herd derestricted in 2013.

Predictor	Odds Ratio	Std. Err.	z	*P* > |z|	Lower 95%CI	Upper 95%CI
**Cat. Index BD lesion**						
**no lesion**	1.000					
**Lesion reported**	1.008	0.091	0.080	0.933	0.844	1.202
**Cat. Index BD reactors**						
**0 ^1^**	1.224	0.115	2.150	0.031	1.018	1.471
**1**	1.000					
**2**	1.176	0.127	1.500	0.133	0.952	1.452
**3**	1.272	0.183	1.670	0.095	0.959	1.686
**4**	1.214	0.236	0.990	0.320	0.829	1.777
**5+**	1.161	0.159	1.090	0.274	0.888	1.518
**Cat. Index BD length (days)**						
**(17–129)**	1.000					
**(130–143)**	1.097	0.126	0.810	0.420	0.876	1.374
**(144–159)**	1.216	0.142	1.680	0.093	0.968	1.528
**(160–229)**	1.059	0.121	0.500	0.616	0.847	1.324
**(230–2918)**	1.489	0.173	3.440	0.001	1.187	1.869
**Cat. Prev. bd**						
**No prev. BD (<5 years)**	1.000					
**Prev. BD**	1.200	0.094	2.340	0.020	1.030	1.399
**log(herd-size)**	1.487	0.059	10.030	<0.001	1.376	1.607
**Herd-type**						
**Beef**	1.000					
**Dairy**	0.874	0.095	−1.240	0.215	0.707	1.081
**Other**	0.550	0.098	−3.340	0.001	0.387	0.781
**Suckler**	0.758	0.070	−3.000	0.003	0.633	0.909
**County**						
**Carlow**	1.000					
**Cavan**	1.565	0.558	1.260	0.209	0.778	3.146
**Clare**	1.322	0.460	0.800	0.423	0.668	2.615
**Cork**	1.217	0.404	0.590	0.555	0.634	2.334
**Donegal**	1.014	0.361	0.040	0.969	0.505	2.036
**Dublin**	3.209	1.635	2.290	0.022	1.182	8.710
**Galway**	0.967	0.340	-0.100	0.924	0.485	1.927
**Kerry**	1.172	0.427	0.440	0.663	0.574	2.393
**Kildare**	1.678	0.689	1.260	0.208	0.750	3.751
**Kilkenny**	1.477	0.523	1.100	0.271	0.738	2.955
**Laois**	1.194	0.457	0.460	0.643	0.564	2.529
**Leitrim**	1.269	0.563	0.540	0.591	0.532	3.029
**Limerick**	1.432	0.515	1.000	0.319	0.707	2.898
**Longford**	0.430	0.204	−1.780	0.075	0.170	1.090
**Louth**	1.465	0.626	0.900	0.371	0.635	3.384
**Mayo**	0.941	0.354	−0.160	0.871	0.451	1.965
**Meath**	1.334	0.468	0.820	0.412	0.670	2.655
**Monaghan**	1.184	0.460	0.440	0.663	0.553	2.536
**Offaly**	1.330	0.496	0.770	0.444	0.641	2.762
**Roscommon**	1.530	0.542	1.200	0.230	0.764	3.063
**Sligo**	1.346	0.514	0.780	0.436	0.637	2.843
**Tipperary**	1.199	0.417	0.520	0.602	0.606	2.372
**Waterford**	0.638	0.270	−1.060	0.288	0.279	1.461
**Wicklow**	2.677	0.970	2.720	0.007	1.317	5.445
**Constant**	0.075	0.028	−6.920	<0.001	0.036	0.156

^1^ Slaughterhouse case.

**Table 2:** General dominance statistics for the final logistic regression modelling of the probability of future breakdown risk during a 5-year follow-up for herds derestricted in 2013.

Outcome: Future Risk	Dominance Statistic	Standardized Domin. Stat.	Ranking
**log_hs**	0.0285	0.4628	1
**County**	0.0153	0.2482	2
**Herd-type**	0.0078	0.1265	3
**Cat. Index BD length (days)**	0.0047	0.0762	4
**Cat. Index BD reactors**	0.0027	0.0445	5
**Cat. Prev. bd**	0.0025	0.0402	6
**Cat. Index BD lesion**	0.0001	0.0015	7

## Data Availability

Data available upon request due to restrictions, e.g., privacy or ethical. The data presented in this study are available upon request from the corresponding authors.

## Supplementary Materials

The following are available online at https://www.mdpi.com/article/10.3390/microorganisms9051004/s1. Table S1: Summary of data on a cohort of herds which was derestricted during 2013. Table S2: Univariable associations between the future risk of breakdown recurrence over a 5 years follow-up period (2013–2018). Figure S1: Box plot of the predicted county variation in risk of future breakdown.
