# Supplementary material for: Future Risk of Bovine Tuberculosis (Mycobacterium bovis) Breakdown in Cattle Herds 2013–2018: A Dominance Analysis Approach
Source: Microorganisms. 2021 May 6;9(5):1004. doi: 10.3390/microorganisms9051004 (PMC8148532; doi:10.3390/microorganisms9051004)
Supplement: Supplementary file 1 [file microorganisms-09-01004-s001.zip › microorganisms-1194905-supplementary.pdf]

Supplementary material

Table S1: Summary of data on a cohort of herds which was derestricted during 2013.

VARIABLE	TOTAL OBS.	OBS	PROP/MEAN	STD. DEV.	MIN	MAX
**FUTURE BD (2013)**						
**0**	4,459	2,990	0.671	0.470	0	1
**1**	4,459	1,469	0.329	0.470	0	1
**INDEX BD LESIONS STATUS**						
**NO LESION**	4,459	2,759	0.619	0.486	0	1
**LESION**	4,459	1,700	0.381	0.486	0	1
**CAT. INDEX BD REACTORS**						
**0**	4,459	1,580	0.354	0.478	0	1
**1**	4,459	1,409	0.316	0.465	0	1
**2**	4,459	644	0.144	0.352	0	1
**3**	4,459	290	0.065	0.247	0	1
**4**	4,459	139	0.031	0.174	0	1
**5+**	4,459	397	0.089	0.285	0	1
**CAT. INDEX BD LENGTH (DAYS)**						
**(17-129)**	4,459	877	0.197	0.398	0	1
**(130-143)**	4,459	891	0.200	0.400	0	1
**(144-159)**	4,459	898	0.201	0.401	0	1
**(160-229)**	4,459	884	0.198	0.399	0	1
**(230-2918)**	4,459	909	0.204	0.403	0	1
**CAT. PREV. BD**						
**NO PREV. BD (<5 YRS)**	4,459	3,414	0.766	0.424	0	1
**PREV. BD**	4,459	1,045	0.234	0.424	0	1
**HERD-TYPE**						
**BEEF**	4,459	924	0.207	0.405	0	1

**DAIRY**	4,459	1,292	0.290	0.454	0	1
**OTHER**	4,459	236	0.053	0.224	0	1
**SUCKLER**	4,459	2,007	0.450	0.498	0	1
**COUNTY**						
**CARLOW**	4,459	50	0.011	0.105	0	1
**CAVAN**	4,459	194	0.044	0.204	0	1
**CLARE**	4,459	297	0.067	0.249	0	1
**CORK**	4,459	596	0.134	0.340	0	1
**DONEGAL**	4,459	257	0.058	0.233	0	1
**DUBLIN**	4,459	28	0.006	0.079	0	1
**GALWAY**	4,459	288	0.065	0.246	0	1
**KERRY**	4,459	172	0.039	0.193	0	1
**KILDARE**	4,459	69	0.015	0.123	0	1
**KILKENNY**	4,459	198	0.044	0.206	0	1
**LAOIS**	4,459	106	0.024	0.152	0	1
**LEITRIM**	4,459	62	0.014	0.117	0	1
**LIMERICK**	4,459	166	0.037	0.189	0	1
**LONGFORD**	4,459	67	0.015	0.122	0	1
**LOUTH**	4,459	57	0.013	0.112	0	1
**MAYO**	4,459	171	0.038	0.192	0	1
**MEATH**	4,459	220	0.049	0.217	0	1
**MONAGHAN**	4,459	104	0.023	0.151	0	1
**OFFALY**	4,459	128	0.029	0.167	0	1
**ROSCOMMON**	4,459	228	0.051	0.220	0	1
**SLIGO**	4,459	134	0.030	0.171	0	1
**TIPPERARY**	4,459	247	0.055	0.229	0	1
**WATERFORD**	4,459	76	0.017	0.129	0	1
**WESTMEATH**	4,459	164	0.037	0.188	0	1
**WEXFORD**	4,459	229	0.051	0.221	0	1
**WICKLOW**	4,459	151	0.034	0.181	0	1

**LOG(HERD-SIZE)**	4,459	4.101	1.165	0	7.212
**HERD-SIZE**	4,459	101.043	102.883	1	1355

Table S2: Univariable associations between the future risk of breakdown recurrence over a 5 year follow-up period (2013-2018).

Variable (outcome = future BD (2013))	N with future BD	(% with future BD)	N	OR	P-value
**Risk factors at animal level**					
**Index BD lesions status**			4459		0.266
**no lesion**	892	32.33		Ref.	
**lesion**	577	33.94		1.075	
**Cat. Index BD reactors**			4459		<0.001
**0**	529	33.48		1.288	
**1**	396	28.11		Ref.	
**2**	226	35.09		1.383	
**3**	108	37.24		1.518	
**4**	52	37.41		1.529	
**5+**	158	39.80		1.691	
**Cat. Index BD length (days)**			4459		<0.001
**(17-129)**	247	28.16		Ref.	
**(130-143)**	269	30.19		1.103	
**(144-159)**	289	32.18		1.210	
**(160-229)**	284	32.13		1.207	
**(230-2918)**	380	41.80		1.832	
**Cat. Prev BD**			4459		<0.001
**No prev. BD (<5 yrs)**	1053	30.84		Ref.	
**Prev. BD**	416	39.81		1.448	
**Herd-type**					<0.001
**Beef**	307	33.23		Ref.	
**Dairy**	548	42.41		1.480	
**Other**	52	22.03		0.568	
**Suckler**	562	28.00		0.782	
**Herd-size**					<0.001
**(<32)**	229	20.95		Ref.	

**(33-69)**	309	27.59		1.438	
**(70-137)**	381	33.84		1.930	
**(138-1355)**	570	49.11		3.640	

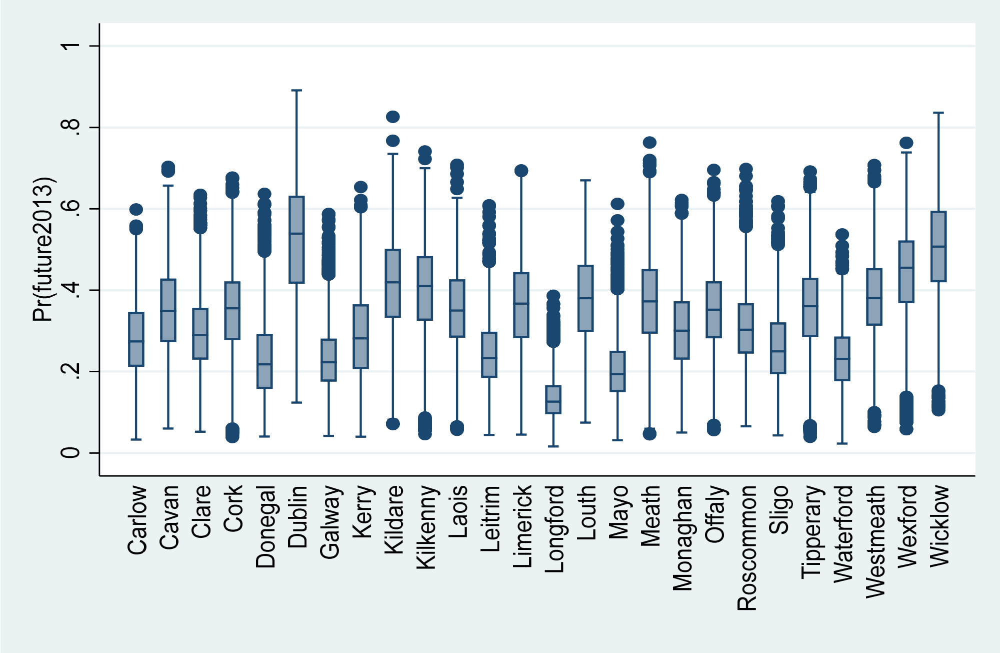

Figure S1: Box plot of the predicted county variation in risk

Supplementary material 1: Full output from a dominance analysis including general, conditional and complete.

Dominance analysis explores the predictors in a model with three parameters, general dominance, conditional dominance, and complete dominance. Complete is the strongest evidence of dominance, and indicates where a predictor is dominant over another predictor in all possible combinations of independent predictors tested (all subsets; Azen and Budescu 2003), i.e. if independent variable *X* has a larger incremental contribution to model fit than independent variable *Y* across all possible comparisons, independent variable *X* "completely dominates" independent variable *Y* (Luchman 2013). Conditional dominance is measured as the mean greater incremental improvement to model fit across a subset of models of size *k*, for which the predictor variable is included. If the average contribution for predictor *X* for this subset of models, is greater than predictor *Y*, then *X* is considered conditionally dominant relative to *Y* (Azen and Budescu 2003). Finally, the weakest evidence of dominance is the general dominance, which is the overall mean of all conditional values, which together add to the fit statistic. Murray and Conner (2009) has recommended the approach as the best for ranking variables, based on a simulation comparison of six competing methods to quantify variable importance.

Total of 127 regressions

Computing conditional dominance

Computing complete dominance

General dominance statistics: Logistic regression
Number of obs = 4459
Overall Fit Statistic = 0.0615

future2013	Dominance Stat.	Standardized Domin. Stat.	Ranking
log_hs	0.0285	0.4628	1
set1	0.0001	0.0015	7
set2	0.0027	0.0445	5
set3	0.0047	0.0762	4
set4	0.0025	0.0402	6
set5	0.0078	0.1265	3
set6	0.0153	0.2482	2

Conditional dominance statistics

	#indepvars: 1	#indepvars: 2	#indepvars: 3	#indepvars: 4	#indepvars: 5
log_hs	0.0400	0.0349	0.0307	0.0272	0.0244
set1	0.0002	0.0002	0.0001	0.0001	0.0000
set2	0.0051	0.0039	0.0031	0.0024	0.0020
set3	0.0078	0.0062	0.0050	0.0042	0.0036
set4	0.0050	0.0037	0.0028	0.0021	0.0016
set5	0.0154	0.0118	0.0089	0.0067	0.0050
set6	0.0208	0.0181	0.0160	0.0144	0.0133

```
         #indepvars:  #indepvars:
                   6            7
log_hs        0.0220       0.0201
  set1        0.0000       0.0000
  set2        0.0016       0.0012
  set3        0.0032       0.0029
  set4        0.0012       0.0010
  set5        0.0038       0.0029
  set6        0.0124       0.0119
------------------------------------------------------------------------------
Complete dominance designation
------------------------------------------------------------------------------

                    dominated?:  dominated?:  dominated?:  dominated?:
                        log_hs         set1         set2         set3
dominates?:log_hs            0            1            1            1
  dominates?:set1           -1            0           -1           -1
  dominates?:set2           -1            1            0           -1
  dominates?:set3           -1            1            1            0
  dominates?:set4           -1            1            0           -1
  dominates?:set5           -1            1            1            0
  dominates?:set6           -1            1            1            1

                    dominated?:  dominated?:  dominated?:
                          set4         set5         set6
dominates?:log_hs            1            1            1
  dominates?:set1           -1           -1           -1
  dominates?:set2            0           -1           -1
  dominates?:set3            1            0           -1
  dominates?:set4            0           -1           -1
  dominates?:set5            1            0           -1
  dominates?:set6            1            1            0
------------------------------------------------------------------------------
```

Strongest dominance designations

log_hs completely dominates set1
set2 completely dominates set1
set3 completely dominates set1
set4 completely dominates set1
set5 completely dominates set1
set6 completely dominates set1
log_hs completely dominates set2
set3 completely dominates set2
set5 completely dominates set2
set6 completely dominates set2
log_hs completely dominates set3
set6 completely dominates set3
log_hs completely dominates set4
set3 completely dominates set4
set5 completely dominates set4
set6 completely dominates set4
log_hs completely dominates set5
set6 completely dominates set5
log_hs completely dominates set6
set5 conditionally dominates set3
set2 conditionally dominates set4

Variables in set1: i.cat_index_lesion

Variables in set2: i.cat_index_reactor
Variables in set3: i.cat_bd_length
Variables in set4: i.cat_prev_bd
Variables in set5: i.herd_type_num
Variables in set6: i.county_num
